# The function of immunomodulation and biomaterials for scaffold in the process of bone defect repair: A review

**DOI:** 10.3389/fbioe.2023.1133995

**Published:** 2023-03-29

**Authors:** Changchao Dong, Gang Tan, Guangyan Zhang, Wei Lin, Guanglin Wang

**Affiliations:** ^1^ Trauma Medical Center, Department of Orthopedics Surgery, West China Hospital, Sichuan University, Chengdu, Sichuan, China; ^2^ Department of Orthopedics, West China School of Public Health and West China Fourth Hospital, Sichuan University, Chengdu, Sichuan, China; ^3^ Department of Respiratory Medicine, The 7th Hospital of Chengdu, Chengdu, Sichuan, China; ^4^ Department of Gynecology, West China Second Hospital, Sichuan University, Chengdu, Sichuan, China; ^5^ Department of Orthopedics, West China Hospital, Orthopedics Research Institute, Sichuan University, Chengdu, Sichuan, China

**Keywords:** bone regeneration, BMSCs, immune cells, biomaterials, interaction

## Abstract

The process of bone regeneration involves the interaction of the skeletal, blood, and immune systems. Bone provides a solid barrier for the origin and development of immune cells in the bone marrow. At the same time, immune cells secrete related factors to feedback on the remodeling of the skeletal system. Pathological or traumatic injury of bone tissue involves changes in blood supply, cell behavior, and cytokine expression. Immune cells and their factors play an essential role in repairing foreign bodies in bone injury or implantation of biomaterials, the clearance of dead cells, and the regeneration of bone tissue. This article reviews the bone regeneration application of the bone tissue repair microenvironment in bone cells and immune cells in the bone marrow and the interaction of materials and immune cells.

## 1 Introduction

Bone is a dynamic, complex system composed of organic and inorganic matter. The inorganic mineral scaffold of bone is endowed with a high degree of plasticity and dynamism of the skeletal tissue through the mosaic of the living cells, the cellular matrix, blood, and the immune system.

The organic and inorganic components occupy approximately 30% and 70% of the bone in the biogenic hierarchical composite matrix (Behzadi et al., 2017). The inorganic component as a bone framework mainly comprises hydroxycarbonated apatite (HA) with a crystal structure (Zhang et al., 2007). Due to its high biocompatibility, engineered HA has been widely used in the biomedical research field of bone regeneration materials. Various cells [Bone marrow mesenchymal stem cells (BMSCs), osteoblasts, osteoclasts, immune cells] embedded in the lacunar steel pipe network of the inorganic framework secrete many functional proteins and extracellular matrix, such as type I collagen fibers, proteoglycan molecules, Osteopontin (OPN), Bone morphogenetic protein (BMP) (Behzadi et al., 2017), Phosphate regulating gene with homologies to endopeptidases on the X chromosome (PHEX) enzyme. The extracellular matrix and signaling molecules constitute the organic components of the bone tissue framework. The delicate “sculpting” of the bone matrix and HA cavity framework by organic functional protein molecules is essential for bone growth and fracture repair.

**FIGURE 1:**
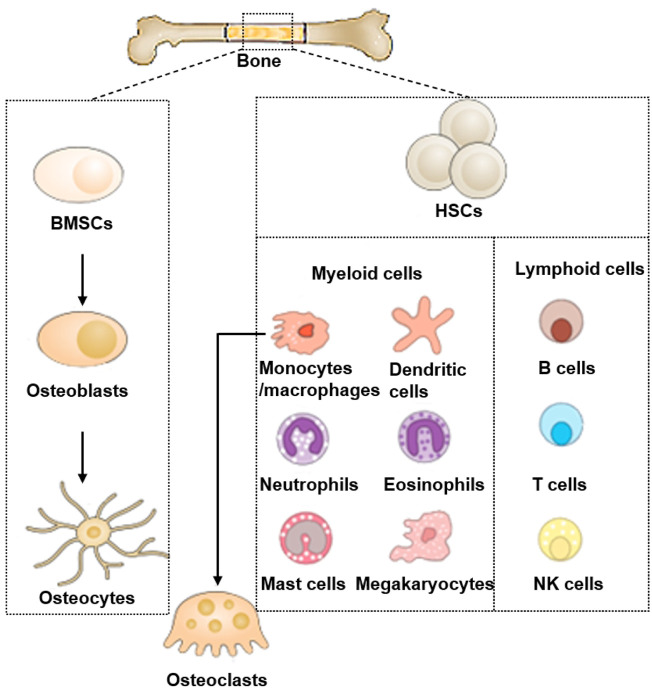
Bone cells and immune cells in bones.

Bone marrow is the principal place of organisms’ hematopoiesis. The multi-polarized bone marrow cavity mainly contains hematopoietic stem cells (HSC), bone marrow and lymphoid progenitor cells, and mature immune cells. The immune cell population includes B, neutrophils, Macrophages, and T cells (Okamoto et al., 2017). Bone and immune cells and other cells in the bone marrow share the same variable microenvironment (Figure 1). In the complex and changeable microenvironment, they interact with each other to perform the “bone immune system” function. The prosperity of osteoimmunology as a self-governed research subject has significantly contributed to the observation of increased bone resorption in every kind of inflammatory skeleton diseases, such as Rheumatoid Arthritis (RA), Osteoporosis (OP), Osteoarthritis (OA), and periodontitis (Tsukasaki and Takayanagi, 2019).

The causes of bone resorption in RA, OP, and OA are not fully elucidated. The scientific community has summarized several reasons. Some views suggest that bone loss occurs due to dysregulation of the microenvironment surrounding these lesions, characterized by increased RANKL expression. Osteocytes, Immune cells (T cells and B cells), and Inflammatory synovial fibroblasts are thought to be important factors in the production of RANKL (Vis et al., 2013; Weber et al., 2019; Fischer and Haffner-Luntzer, 2022; Komatsu and Takayanagi, 2022). In addition, it has been reported that the increased expression of inflammatory factors (IL-6, TNF-α, etc.) is also an important cause of bone loss. On the one hand, these inflammatory factors assist RANKL in inducing osteoclast formation. On the other hand, it regulates Dickkopf-1 (Dkk-1) and sclerostin (SOST) to inhibit the Wnt signaling pathway for reducing osteoblast diffiration (Hickman and Pierson, 2016; Weber et al., 2019; Komatsu and Takayanagi, 2022). The researchers have designed a variety of excellent biomaterials to interfere with the inflammatory process in three diseases. Strontium-substituted bioactive glass (SR-BG) significantly inhibited bone loss by inhibiting p38 and NF-ΚB pathways downstream of RANKL (Huang et al., 2020). Chitosan, similar to the structure of biological tissue glycosaminoglycans, has made polymer and derivative forms that improve the oral utilization of the drugs of Risedronate sodium and Alendronate sodium to achieve good effects in inhibiting osteoclasts (Rahimi et al., 2022). Bioactive particles such as HAMA microspheres (Li et al., 2022), modified liposomes (Feng et al., 2020), and chitosan-modified nanoparticles (Shi et al., 2018) can be used as gene delivery vectors (IL-6-siRNA, TNF-α, etc.) to treat arthritis and bone loss by decreasing the expression of inflammatory factors in RA and OA.

Unlike the self-repair of minor fractures, the ability to self-heal from bone damage with large defects may be limited. The migration and differentiation of osteoblast precursor cells is the key process of bone injury repair, but it is difficult for these cells to find the attachment site in the condition of large bone injury (Abbasi et al., 2020). Therefore, applying biomaterials provides a promising solution for this kind of damage. The application of Osteogenic glue and artificial periosteum has successfully repaired comminuted fracture and large bone defect models (Wu et al., 2020; Xin et al., 2020; Tang et al., 2021). These successful cases of preclinical bioactive materials will provide references for developing more clinical biomaterials applications.

The application of autologous bone, allogeneic bone, or bone substitute material has been a common strategy in the surgical treatment of bone defects. The generation of blood vessels and the invasion of osteoblast precursor cells are the keys to the successful treatment of bone defects. Insufficient vascularization and poorly cast bone matrix may lead to cell necrosis or detachment of bone substitutes (Gotz et al., 2012; Zheng et al., 2022). Thus, the coupling of angiogenesis and osteogenesis is the key to the successful repair of bone defects. Autocrine and paracrine factors determine the interaction between osteogenesis and angiogenesis. Factors such as Vascular endothelial growth factor (VEGF), Hypoxia-inducible factor (HIF), and Osteopontinntin can act bidirectionally on endothelium and osteoblasts (Gotz et al., 2012). It is beneficial for angiogenesis and new bone formation. Materials biologists have combined these factors to design a variety of advanced biomaterials that are different from conventional implantation therapy. Cross-linked engineered VEGF (TG-VEGF) modified fibrin matrix significantly promoted early vascular invasion and osteogenic differentiation of critical-size skull defects (Burger et al., 2022). In addition, exosomes from BMSCs carrying HIF-1α load onto traditional β-TCP scaffolds contribute to new bone regeneration and Neovascularization in critical-size bone defects (Ying et al., 2020). With the further study of bone immunology, the combination of immune factors has been introduced into new bioactive scaffolds for osteogenesis and angiogenesis. 3D-printed PCL scaffolds of Plla electrospun microfiber (3D-M-EF) and nanofiber (3D-N-EF) composites have the ability to modulate macrophage M2 polarization, and VEGF and BMP-2 are secreted by M2 to promote angiogenesis and osteogenesis of rat skull defect (Liu et al., 2021). Various metal particles have been studied for the interaction between immune cells and bone regeneration (Zhao et al., 2021a; Wan et al., 2022; Wu et al., 2022; Li et al., 2023). Therefore, the intervention of immune microenvironment materials will open a new chapter for the repair strategy of bone regeneration.

Innovation in biomedical technology will provide a better basis for bone defect repair, 3D printing technology has been successfully applied to materials science, optics, Robotics and chemistry, and other scientific fields. With the application of 3D printing in biomedicine, it has been successfully applied to print living cells or specific tissues, but there are some challenges in the application of 3D printing technology. The choice of printing ink determines its Biocompatibility and cellular distribution. In addition, changes in forced shear and photothermal induction during 3D printing may have a greater impact on cell viability. The biggest problem with 3D printing is that it cannot respond to the biological needs of the tissue. These reasons led to the development of 4D bio-printing technology. Materials scientists have studied 4D bio-printing techniques that are programmable, multi-material combinatorial, and bio-tissue responsive to adaptation. Although the 4D printing technology has great challenges in easy manufacturing, economy, and extensibility, its considerable biomedical application prospect is worth paying attention to (Osouli-Bostanabad et al., 2022).

Various immune cells (T, B, Dc) regulate bone homeostasis. Moreover, Various molecules (cytokines, chemokines, and signaling molecules) in the bone microenvironment are regulated and utilized by various cells in the bone marrow (Okamoto et al., 2017). In addition, A growing body of evidence suggests that bone and immune cells regulate each other to regulate hematopoietic function further. Regarding the evolutionary perspective, the simultaneous development of acquired immunity and the skeletal system maybe lead to shared mechanisms and close the effect of skeletal cells and immune cells. Further, various cells and molecules interact with each other to regulate their development.

The interaction between the immune system and the biological material relies on the relationship between the graft and the surrounding tissues, mediating processes such as cell-specific innate defense and adaptive immune response. It is increasingly evident that macrophages residing in tissues or macrophages recruited from other regions play different parts in the healing stage, and the same material implanted in different parts will cause different effects (Reid et al., 2015). However, a common feature of biomaterials is to induce unfavorable immune responses, leading to excessive inflammation, tissue destruction, healing disorders, fibrotic encapsulation, and even isolation and rejection of medical materials. Resolving these side effects requires further exploration of the interaction between the material and the surrounding tissue microenvironment, which is both a challenge and an innovation.

## 2 Effects of the immune system on bone marrow cells

### 2.1 Bone marrow cells

**FIGURE 2:**
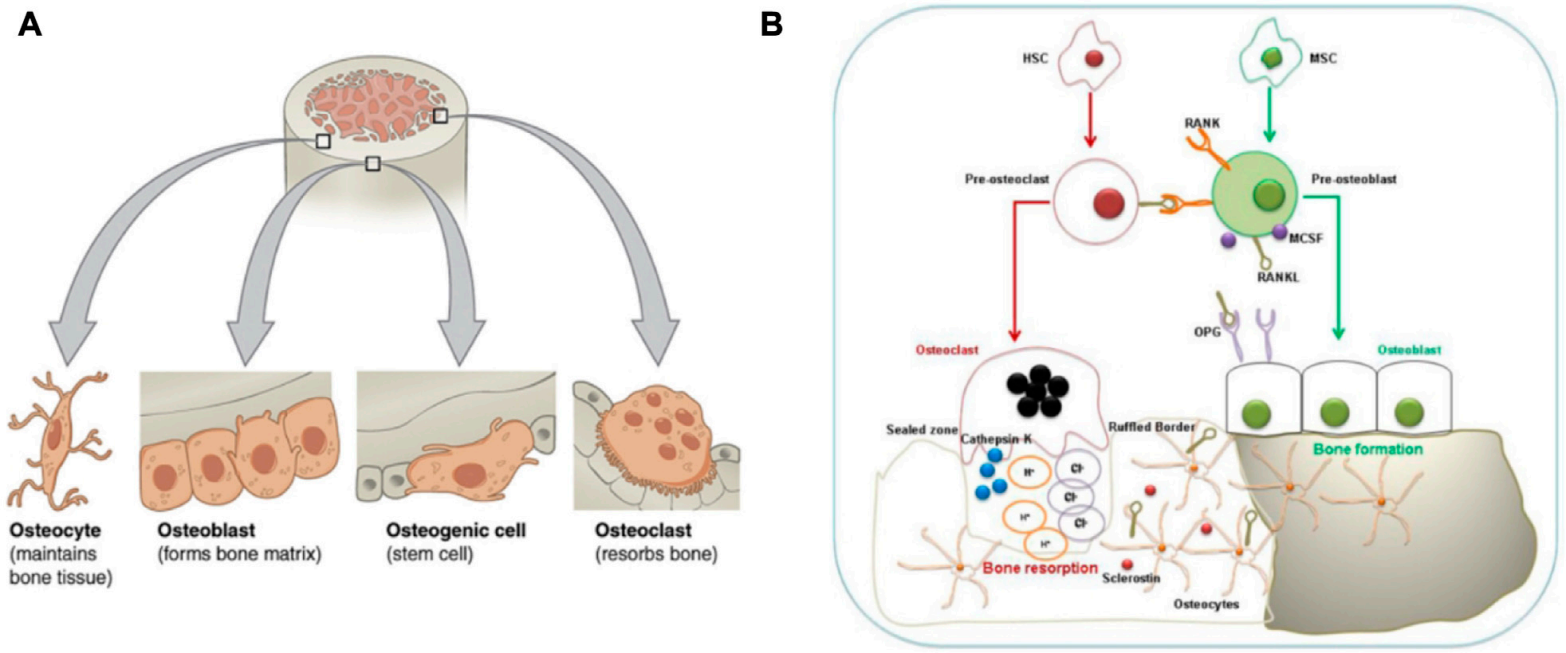
Bone remodeling and its involved cells. **(A)**:Four types of cells are found within bone tissue. **(B)**: Interaction between Osteoblasts-Osteoclasts-Osteocytes during bone remodeling. The graph was reprinted with permission from (Behzadi et al., 2017) and (Dar et al., 2018).

Continuous bone remodeling occurs in the bones throughout the organism’s life cycle. Osteoblasts, osteoclasts, and osteocytes work together to complete the dynamic bone remodeling of bones (Figure 2) (Naik and Wala, 2013; Behzadi et al., 2017). Bone remodeling involves the Interplay of the time and space of osteoblast, which leads to bone formation, and osteoclast, which leads to bone resorption (Naik and Wala, 2013).

Osteoblasts are clusters of bone marrow mesenchymal stem cells covering the bone’s surface. They are metabolically active and can synthesize and secret collagen and non-collagen bone matrix proteins deposited between bone cells and the surface. These uncalcified deposits are called osteoid, and the osteoid mineralization cycle is 10 days. BMSCs can differentiate into osteoblasts. The process involves the expression of Runx2 and Osterix transcription factors based on external stimuli such as Parathyroid hormone (PTH), Prostaglandin E 2 (PGE2), and Insulin-like Growth Factor (IGF) (Tsukasaki and Takayanagi, 2019). Moreover, BMP-2 and Wnt signal pathways are responsible for osteoblast or BMSCs differentiation.

Osteocytes connect through a small tube connected to the bone surface and form a network, the dense signal communication path in the bones. Osteocytes are derived from bone osteoblasts, then wrapped in the bone matrix. However, Osteocytes begin to express some specific genes, but osteoblasts do not express these genes. Sclerostin is a product of osteocytes, which can bind to low-density lipoprotein receptor-related protein (LRP) and inhibit the Wnt signal pathway to reduce bone formation (Poole et al., 2005). Function Sclerostin (SOST) gene encoding sclerostin mutations results in an increased bone mass loss in humans, called sclerosis.

Osteoclasts originate from HSCs. HSCs first develop into monocytes. Then monocytes develop into mature osteoclasts under the stimulation of related factors. Osteoclast production requires macrophage colony-stimulating factor (M-CSF) and receptor activator of nuclear factor-κ B ligand (RANKL) as induction signals. Osteogenic precursor cells, stromal cells, and synovial endothelial cells express M-CSF and RANKL to induce osteoclastogenesis. Along with BMMs, they differentiate and form mature osteoclasts. At this time, osteoclasts also begin to express specific genes and fuse. Moreover, RANKL is expressed on preosteoblasts and activated T cells (Wada et al., 2006; McInnes and Schett, 2007). RANKL combines its receptor RANK with pro-osteoclast cells, which is grave for the differentiation of osteoclasts and their bone resorption capacity. The interaction between RANKL and RANK is regulated by the RANKL competitive receptor OPG, which inhibits osteoclast production *in vitro* and *in vivo*. In addition to the RANKL and RANK affecting each other, other key pro-osteoclast signaling pathways depend on trigger receptors (TREM) expressed on bone marrow cells. The OSCAR and the tyrosine kinase DAP12 interact with triggering receptors expressed on myeloid cells (TREM), which strongly promotes osteoclastogenesis (Barrow et al., 2011).

### 2.2 Immune system

**FIGURE 3:**
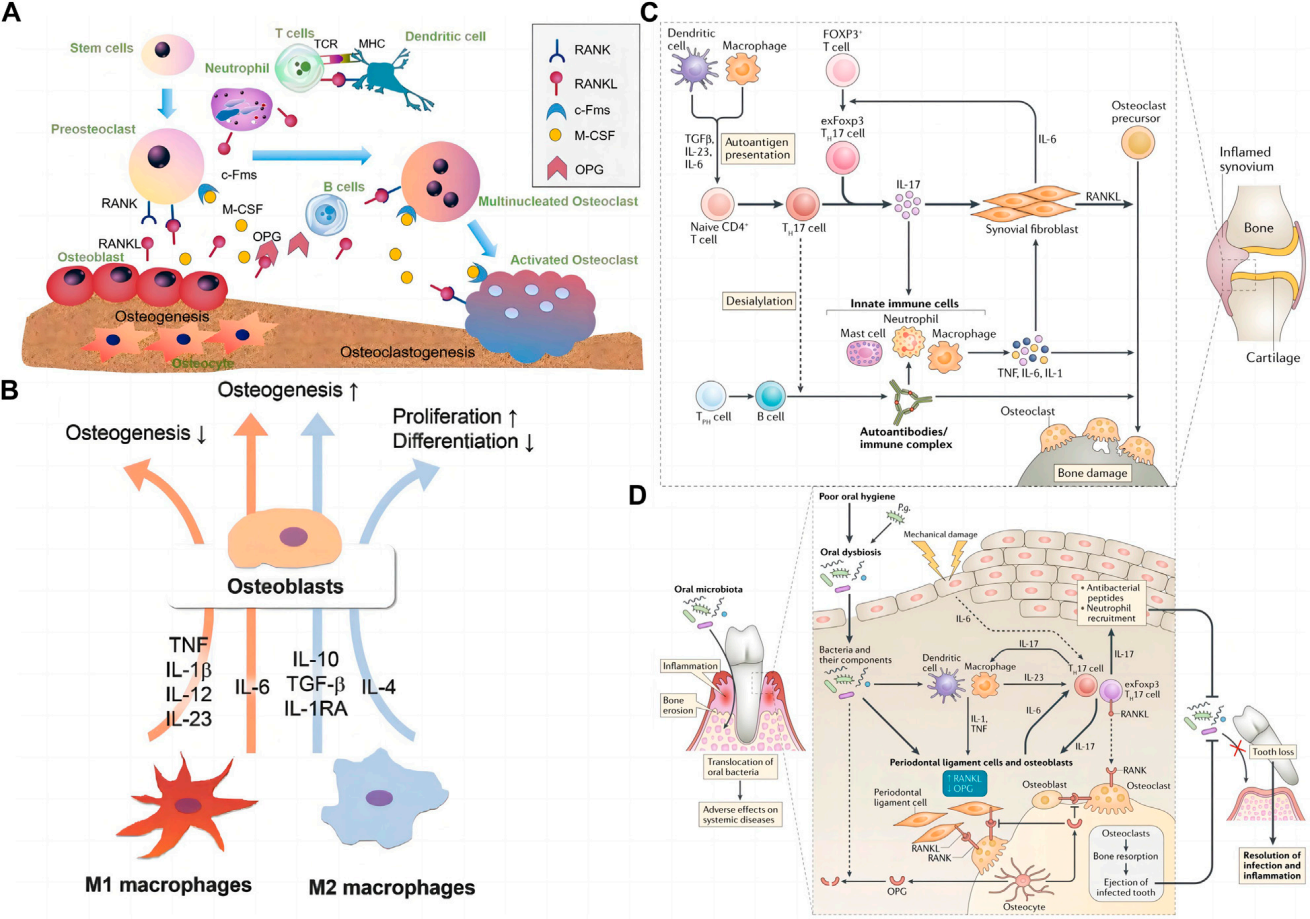
The interaction of the skeletal system and the immune system. **(A)** Interaction between skeletal and immune cells by secreting related factors, the graph was reprinted with permission from (Xie et al., 2020). **(B)** M1 and M2 interact with osteoblasts through cytokines such as IL-6, IL-23, IL-10, and TGF-β. The graph was reprinted with permission from (Lee et al., 2019). **(C)** T cells, macrophages, and neutrophils’ role in rheumatoid arthritis-induced bone erosion, the graph was reprinted with permission from (Tsukasaki and Takayanagi, 2019). **(D)** The role of T cells, macrophages, and DCs, in bacterial periodontitis-induced bone erosion, the graph was reprinted with permission from (Tsukasaki and Takayanagi, 2019).

The immune system is essential for organisms to defend against foreign objects. In addition, the invasion of pathogens can also stimulate the evolution of the immune system. The body’s adaptability and innate immune response participate in this process. As mentioned above, both immune and bone cells are present in bone. Meanwhile, the two systems influence each other (Lee et al., 2019) (Figure 3A). Bone cells and their surrounding environment secrete chemokines [Macrophage chemoattractant protein-1 (MCP-1), Stromal cell-derived factor 1 (SDF-1), etc.] that attract the arrival of immune cells, which subsequently secrete cytokines (RANKL, IL-1, IL-6, IL-10, TNF-α, etc.) that affect the development of bone cells. Thus, basic and clinical researchers explore the interaction between immune cells and their factors with bone cells (Naik and Wala, 2013). Although immune cells are various, they originate from a common ancestor-HSCs. HSCs are finally differentiated into T cells, B cells, macrophages, NK cells, and neutrophils through pluripotent stem cells, myeloid stem cells, and lymphoid stem cells and enter different tissues or organs with the bloodstream to exert their physiological functions further.

#### 2.2.1 Macrophages

Macrophages belong to the mononuclear phagocytic cell system. They also originate from HSCs. In the bone marrow, HSCs develop into monocytes through immature monocytes, enter connective tissues and other organs with the bloodstream, and then turn into macrophages. The body’s homeostasis processes include inflammation, foreign body removal, and bone tissue repair (Figure 3B) based on macrophages’ differentiation and function (Xie et al., 2020).

Macrophages are a class of highly plastic immune cells whose main function is to play the role of phagocytosis. It engulfs foreign bodies (bacteria, viruses, nanoparticles, etc.) that invade the organism and engulfs the dead and apoptotic cells it produces. Phagocytosis of different substances by macrophages produces different body responses. Macrophages form the “classical activated” M1 phenotype after phagocytosis of pathogens and dead cells, which mainly plays a pro-inflammatory role, whereas, when macrophages engulf apoptotic cells, they mainly form “alternatively activated” M2 phenotype, which promotes tissue repair by secreting repair factors such as IL-10 and TGF-β (Xie et al., 2020; Schlundt et al., 2021). In bone defect repair, plasma proteins and foreign bodies attract macrophages to the defect. In the early phase of inflammation, macrophages transform into M1 macrophages, which in the early phase play a clearing role of foreign bodies and dead cells, and subsequently, the transformation of macrophages into M2 type initiates the process of bone tissue regeneration. Therefore, the ratio of M1/M2 macrophages is a key process in the transition from inflammation to regeneration (Zhao et al., 2021b).

Compared with monocytes, multi-nucleated cells formed by the fusion of macrophages have a phagocytic affinity for significant substances. The ability of macrophages puts them at the center of the evolutionary and functional roles of the bones and immune system because monocytes can form granulomas and other giant cells in inflammation regions, and they can also fuse in bones to form large multi-nucleated cells called osteoclasts. The decision to proceed with osteoclast forming is only controlled by the local cells factor environment of RANKL (Dar et al., 2018). Part of macrophages participate in the fight against harmful damage at the site of tissue inflammation; they can perform multiple functions. Inflammatory macrophages can clear apoptotic cells, proliferate and resident matrix, infiltrate white blood cells, and kill parenchymal cells (Naik and Wala, 2013). In addition, heterotopic bone formation in fibrodysplasia ossificans progressiva (FOP) patients is thought to be triggered by inflammation such as macrophages and mast cells l (Convente et al., 2018).

#### 2.2.2 T cells

T cells are lymphoid stem cells derived from the bone marrow. They are a group of cells that develop and mature after migrating into the thymus. It mainly includes CD4 T cells and CD8 T cells. CD4 T cells mainly develop into Th cells. Naïve Th cells can transform into subgroups according to the antigens and cytokine stimuli they receive during activation. And then, Th cells can be well divided into four subgroups, namely, Th1, Th2, Treg, and Th17 cells. Unactivated Th cells can inhibit the formation of osteoclasts *in vitro* (Toraldo et al., 2003) or *in vivo* (Li et al., 2007). Under steady-state conditions, Th cells cannot secrete RANKL (Dar et al., 2018). In contrast, the activation of T cells under inflammatory conditions can lead to increased production of RANKL and Tumor Necrosis Factor-α (TNF-α), which promote osteoclast production and bone loss under kinds of inflammatory and autoimmune conditions (Colucci et al., 2004), such as periodontitis (Brunetti et al., 2005), cancer (Colucci et al., 2004) and osteoporosis (Faienza et al., 2009). However, not all kinds of T cells can activate osteoclasts. It is reported that CD8^+^ T cells have osteoprotective effects. CD8^+^ T cells can inhibit osteoclastogenesis by secreting OPG (Dar et al., 2018).

Th1 cells can secret Interleukin-2 (IL-2), Interleukin-12 (IL-12), interferon-γ (IFN-γ), and TNF-α to participate in the clearance of pathogens. Moreover, Th2 cells can secret IL-4, IL-5, IL-6, IL-9, and IL-13 cytokines to participate in the clearance of parasitic infections, extracellular microorganisms, and allergic diseases. It is now known that Th1 and Th2 cells can inhibit the formation of osteoclasts in different populations by secreting their iconic cytokines IFN-γ and IL-4, respectively (Harrington et al., 2006). In the past decade, two new CD4^+^ Th cell subgroups have been reported, which were Th17 cells (Harrington et al., 2006) and Treg cells. Rorγt transcription factor defines Th17 cells, and the Foxp3 transcription factor specifically defines Treg cells. They have been confirmed that these two kinds of Th cells are the primary T cells that regulate osteoclast production (Dar et al., 2018).

Transforming growth factor-β (TGF-β), IL-6, and inflammatory stimuli induce Naïve Th cells to transform into Th17 cells. Th17 cells produce IL-17, the hallmark factor of Th17 cells, as well as IL-22, IL-26, and IFN-γ (Harrington et al., 2006; Gao et al., 2007). Th17 cells induce osteoclastogenesis by producing IL-17, a common factor that promotes osteoclastogenesis by inducing RANKL (Adamopoulos et al., 2010). However, IL-17 can promote osteoclast production through direct and indirect action, mainly through two inflammatory factors, TNFα and IL-1 (Dar et al., 2018). These cytokines in cells enhance the expression of RANKL that support osteoclastogenesis, activating pro-osteoclast through the RANKL-rank signal pathway. In the occurrence of various bone diseases, Th17 cells are involved, such as RA (Figure 3C) (Tsukasaki and Takayanagi, 2019), periodontitis (Figure 3D) (Tsukasaki and Takayanagi, 2019), osteoporosis, and OA (Maddur et al., 2012).

It has been reported that a specific type of TH17 cells-exFoxp3TH17 cells, compared with TH17 cells, can activate osteoclasts. Treg cells express the specific molecule Forkhead Box Protein P3 (FOXP3). In arthritis animals, when CD25loFoxp3+T cells lose FOXP3 expression and start to transform into TH17 cells, inflammation stimulates IL-6 produced by synovial cells. Importantly, Foxp3+IL-17+T cells are particularly prominent in the synovial tissue of patients with active RA but not detected in the synovial fluid of inactive patients, which has been observed in membrane tissue. It indicates the pathogenic effect of exFoxp3TH17 cells in RA. Furthermore, the increase of IL-6 secreted by synovial fibroblasts contributes to the production of exFoxp3TH17 cells. In turn, the increase of IL-17 can stimulate the production of CCL20, IL-6, granulocyte-macrophage colony-stimulating factor (GM-CSF), and RANKL in synovial tissue, thereby exacerbating local inflammation and joint destruction (Hirota et al., 2018). Therefore, Bone-destructive T cells and synovial tissue’s malignant circulation mechanism is an effective research field for treating bone diseases.

CD4^+^ Treg cells and TH17 cells have opposite effects. CD4^+^ Treg cells can suppress osteoclast differentiation and function by secreting a kind of cytokines which include TGF-β and IL-4 cytokines, in a cytokine-dependent manner. Recently, In the ovariectomized osteoporosis model, by producing IL-10 and TGF-β1, Treg cells can reduce osteoclast differentiation and bone resorption. Another reported mechanism is that Treg cells can produce Cytotoxic T Lymphocyte-Associated Antigen-4 (CTLA-4) to control immune function, bind to CD80/CD86 on pre-osteoclast cells, and inhibit inflammation (Dar et al., 2018). According to reports, oral probiotic *Lactobacillus* rhamnosus GG can stimulate osteogenesis through the Wnt process of Treg cells acting on CD8+T cells. In addition, in the process of bone healing, it is shown that Vγ6+γδ T cells produce IL-17 and promote bone formation (Ono et al., 2016). These findings demonstrate the critical role of immune cells in calcification formation.

NK T cells can clear viral infections, transformations, or abnormal cells. Activating natural NK T (iNK T) can cause active osteoclasts. In the synovial fluid of a confirmed RA patient, NK T cells account for 20% of all lymphocytes. Moreover, CD56bright NK T cell subgroups upregulate many adhesion molecules and chemokine receptors, which can help preferentially recruit NK T cells to RA In the patient’s inflamed synovium. NK T cells also produce M-CSF and RANKL, which are beneficial for inducing osteoclastogenesis (Dar et al., 2018).

#### 2.2.3 B cells

The role of B cells with other immune cells and bone cells is complex and diverse (Dar et al., 2018). B cells are differentiated from HSCs in the skeleton. The osteoblast cells, which are in the skeleton niche, can support HSC and B cell differentiation. B cells play a vital function in the pathogenesis of RA. High serum RA factor and Anti-Citrullinated Protein Ab (ACPA) are related to the course of aggressive bone injury. Immune complexes, including ACPA, have been seen to enhance osteoclast differentiation by activating Fcγ receptors on Osteoclast progenitor cells (OPCs) (Harre et al., 2015). It is a rare immune complex function separated from the host’s defense environment. ACPA also accelerates osteoclast production through its Fab fragments. The citrulline epitopes on OPCs and osteoclasts bind to ACPA to stimulate TNF and IL-8 production, thereby enhancing osteoclast production in an autocrine manner (Krishnamurthy et al., 2016).

It has been reported that both B cell and B cell-derived plasma cells can express RANKL, decoy receptor 3 (DcR3), or IL-7 secretion (a potent bone resorption stimulator), which indirectly regulates osteoclastogenesis. Plasma cells derived from the tumor in patients with multiple myeloma can reduce bone formation by producing cellular molecules (like dickkopf-related protein 1 and sclerostin) (Dar et al., 2018). The lack of estrogen can cause the increase of B lymphocytes, and the corresponding treatment can inhibit the increase of B lymphocytes. Interestingly, the immature B cell population that expresses B220 can even be transdifferentiated into the osteoclast differentiation pathway under *in vitro* conditions, which provides a source of osteoclast precursors for the bone loss caused by ovariectomy. It has been shown that the RANKL expression of B220+ cells is enhanced in ovariectomized mice. In addition, it has been reported that postmenopausal mammalian-derived B cells from the bone can secrete RANKL, and B cells have a positive role in OP, like OPG, whose lack can lead to increased osteoclast production. In addition, the effect of B and T cells can regulate the forming of many bone cell factors because B cells inhibit osteoclast production by Th1 cell activation but promote osteoclasts when it comes to Th2 cell s activation (Li et al., 2007). The two types of cells can also influence the OPG from B lineage cells through the CD40/CD40L interaction pathway, which limits basal bone resorption *in vivo* (Yamamoto et al., 2019).

#### 2.2.4 Dendritic cells (DCs)

DCs are highly effective APCs and are key in treating pathogens and tumors mediated by cellular immunity. Under the action of immune cell CD4 T cells, mouse CD11c^+^DCs can successfully transform into functional osteoclasts and induce osteoclast-mediated bone loss (Dar et al., 2018). These findings suggest the strong effect of CD11c^+^DC subpopulations in osteoclast forming. The RANK-RANKL signaling pathway also plays a role in the osteoclast transformation process of DCs (Tucci et al., 2011). In addition, mature DC can promote Th17 cells to express IL-17, enhancing osteoclastogenesis (Dhodapkar et al., 2008).

#### 2.2.5 Neutrophils

Neutrophils are derived from hematopoietic stem cells of bone marrow. After differentiation and development in bone marrow, neutrophils enter the blood or tissues. In the start stages of inflammation caused by bacterial infections, environmental exposures, and certain cancers, neutrophils are essential to innate immunity. In addition, high concentrations of RANKL are also derived from the neutrophils of the mammalian immune system, thereby activating bone loss (Dar et al., 2018). It has been further reported that neutrophils can regulate osteoblast function, leading to increased bone resorption (Brunetti et al., 2013).

### 2.3 Bone marrow cells regulation of HSCs and immune cells

The balance of HSCs in the bone is inseparable from the role of cytokines like Stem cell factor and chemokine C-X-C ligand12 (CXCL12). Since CXCL12 is produced in the body by abundant reticulocytes and leptin receptor-positive osteoblasts or osteoprogenitor cells instead of mature osteocytes, it is an essential source of these niche factors regulated by HSCs (Zhou et al., 2014). Immune cells originate from the bone. Osteoblasts modulate the differentiation of immune cells beyond doubt. For example, it has been reported that consuming osteoblasts’ CXCL12 reduces the number of B lymphoid progenitor cells in the bone marrow. Moreover, osteoblasts express Notch ligand delta-like 4, which helps support the growth of T cell progenitor cells (Yu et al., 2015). Under certain conditions, osteoblasts may also have different roles in the process of hematopoiesis. For example, bone cell-derived Dickkopf-1 (DKK1) directly promotes hematopoietic reconstitution after bone marrow suppression by inhibiting HSCs senescence and indirectly promotes hematopoietic reconstitution by inducing the secretion of bone epidermal growth factor. It is reported that bone marrow endothelial cells (Himburg et al., 2017) and osteoblasts can regulate red blood cell production. Activated osteoblasts can weaken the development of leukemia (Krevvata et al., 2014). Activated CD4^+^ T cells reduce the bone formation of osteoblasts and lead to imperfect B-cell lymphocyte production, which proves an interaction between bone and immune cells (Shono et al., 2010).

The osteoclast bone resorption produces the bone marrow cavity where the secretion of osteoblasts, bone cells, and other cells are attached. Due to the abnormal balance of osteoclastic and osteogenic differentiation in osteosclerotic animals, there is insufficient space in the bone marrow to support the differentiation of hematopoietic cells, ultimately leading to abnormal extramedullary hematopoiesis in the spleen and liver. This abnormal process is detrimental to the formation of immune cell differentiation and function (Okamoto et al., 2017). Patients with bone sclerosis may appear with anemia and infections due to abnormal hematopoietic function (Sreehari et al., 2011). Therefore, the cavity microenvironment formed by osteoclast resorption is necessary for normal functional hematopoiesis. Moreover, osteoclasts play a certain role in the mobilization of HSCs.

## 3 Interaction of materials and immune cells

### 3.1 Biomaterials

With the development of biomaterials, their status in medical care is getting higher and higher. At the same time, many new disciplines have been produced, and huge development and applications have been achieved, such as medical implants, drug delivery, tissue engineering, and immunotherapy (Vishwakarma et al., 2016; Roseti et al., 2017). Biomaterials include a series of compounds with very different functions and structural characteristics, from macromolecules taken from nature to completely synthetic nanoparticles. Biomaterials should be mechanically resilient, Biocompatibility, or capable of degradation in the time required for bone cell colonization and mineralization. Biocompatibility is the primary target of choice for bone implant materials. Moreover, bone is a load-bearing organ with certain mechanical strength, and biomaterials with the same Young’s modulus as bone are highly sought after. For repairable bone injuries, biomaterials are generally selected to be biodegradable at a rate comparable to the bone healing cycle (Borchers and Pieler, 2010; Gorejová et al., 2018; Zhu et al., 2022).

There are many classification methods for biomaterials, which are classified into natural polymers and synthetic polymers according to polymers. The most commonly used tissue regeneration materials, especially orthopedics materials, like synthetic polymers, ceramics, and natural polymers (Mariani et al., 2019). Ceramic materials (glass, alumina, zirconia, calcium phosphate) are mainly used in orthopedics, dental implants, and bone filling. They have the characteristics of low elasticity, high-temperature resistance/hardness, and high brittleness. The chemical structure and physical properties similar to natural bone tissue show good biocompatibility (Mariani et al., 2019). Synthetic polymers as promising biomaterials for bone tissue engineering research and use. Moreover, natural polymers similar to natural ECM make them great biocompatible. They combine synthetic and natural polymers’ advantages, including immunological properties. The morphology and physical properties of the materials have different effects on cell behavior (Table 1). With the development of material technology, biodegradable biomaterials are particularly popular in bone tissue engineering.

**TABLE 1:** The effects of material properties on cell behavior.

Biomaterials	Features	Function	Ref
PCL/P3ANA-RGD nanofibers	Higher surface area and better mechanical properties	Favored cell attachment, proliferation, and osteogenic activity	Guler et al. (2017)
Laminin–PHB fibrous	Simulation of surface morphology and chemical composition of extracellular matrix (ECM)	The adhesion and proliferation of nerve cells are promoted	Sangsanoh et al. (2017)
PEDOT/Cs/Gel scaffolds	The incorporation of PEDOT on the scaffold increased the electrical conductivity, hydrophilicity, mechanical properties, and thermal stability	The adhesion and proliferation of neuron-like cells are promoted	Wang et al. (2017)
PLGA-GNP fibers	Poly (lactide-co-glycolide) (PLGA) can effectively control the loading of gold nanoparticles (GNP)	Osteogenic differentiation of human adipose-derived stem cells is increased	Lee et al. (2018)
β-Tricalcium phosphate (TCP) scaffolds decorated with polydopamine nanoparticles PDA-NPs	Affinity for a variety of proteins and peptides	Excellent osteoinductivity and bone-regeneration	Wang et al. (2016)
Cylindrical PVA hydrogel	Stiffness gradient	Investigate the effects of substrate stiffness on stem cell differentiation into specific cell types	Kim et al. (2015)
Randomly-oriented fibrous scaffolds	Different topographic materials utilized as bio-scaffolds *in vivo*	Cytoskeletal tension release abrogated the divergent differentiation pathways on different substrate topography	Yin et al. (2015)

Biomaterial polymers include natural and synthetic biomaterial polymers (Wei et al., 2020) (Table 2).

**TABLE 2:** Classification of biological materials.

Natural biomaterial polymers	Synthetic biomaterial polymers
Collagen	PCL, PGA
Chitosan	PLGA, PHB
Fibrin	Biodegradable Ceramics
Silk Fibroin	Bioactive glasses and biodegradable metals

The most common natural biomaterial polymers are collagen, chitosan, fibrin, and silk fibroin, which have good histocompatibility and low immunogenicity due to their close similarity to animal tissue components. Decomposition products are also the best raw materials for the body’s biosynthesis, so they are prevalent in tissue engineering. However, these single materials have poor mechanical strength and rapid degradation, and their application in bone tissue is limited. Therefore, these materials are often combined with bioceramics and synthetic polymers.

Synthetic biomaterial polymers such as poly(ε-caprolactone) (PCL), polylactic acid, PGA, copolymer PLGA, poly(3-hydroxybutyrate) PHB, biodegradable ceramics, bioactive glasses, and biodegradable metal materials, which are biocompatible and have a controllable rate of degradation, and their degradation products have no toxic effects on tissues *in vivo*. Moreover, manual control of design and synthesis parameters can produce polymers with better mechanical properties.

### 3.2 Immune cell responses to biomaterial implants

**FIGURE 4:**
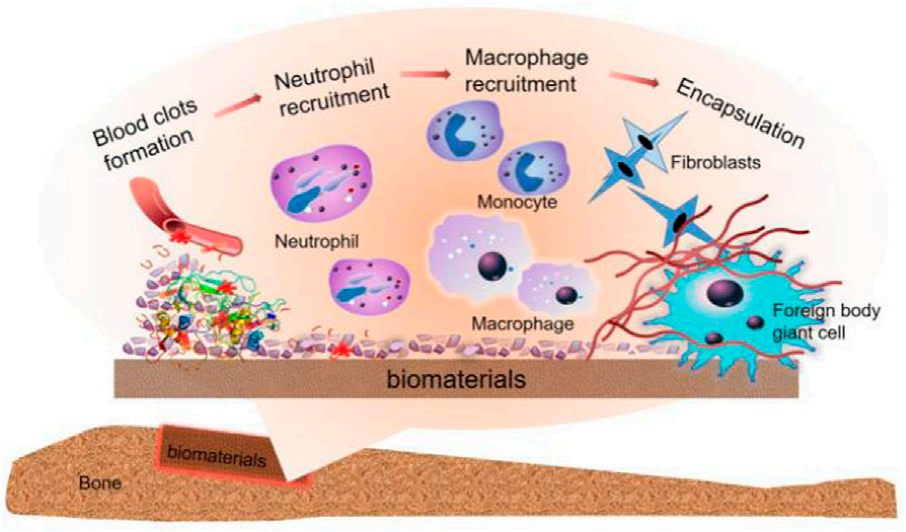
Immune cascade after bone implant material, the graph was reprinted with permission from (Xie et al., 2020).

Preparing the materials mentioned above into biodegradable composite materials is the focus of current research. These composites possess excellent biocompatibility, osteoconductivity, mechanical strength, and osteogenic properties. Meanwhile, these composite materials have become the most promising materials in bone defect repair with the help of new preparation technologies that have emerged in recent years. A systematic understanding of the specific processes of materials in bone regeneration in the emergency immune response is the practical basis for designing composite materials for immune cell regulation (Xie et al., 2020) (Figure 4).

The implant is placed a second later, and blood from the damaged blood vessel surrounds the biomaterial and begins interacting with the graft. And then, Plasma contents of the body, including proteins (fibrinogen, albumin, vitronectin, fibronectin, gamma globulin), attach to the implant surface quickly and autonomously (Wilson et al., 2005). These are affected by the process, quantity, composition, and conformational changes of adsorbed molecules of biological materials.

The clot formed by blood exudate defines the temporary matrix surrounding the biological material (Ekdahl et al., 2011) and is cleaved into fibrin by thrombin. Moreover, the complement protein is activated when it comes into contact with biological materials to support platelet adhesion and activation. The recruitment and adhesion of sufficient immune cells are seduced by abundant pro-inflammatory cytokines, chemokines, and growth factors (Anderson et al., 2008), and with the formation of the temporary matrix, acute and chronic inflammation also follows.

The chemokine produced by the host cell or damaged tissue induces activated neutrophils to be recruited from the peripheral blood and adhere to the implantation site (*via* β2 integrin). It also attempts to produce engulfing proteolytic enzymes and ROS to destroy/degrade biological materials (Grandjean-Laquerriere et al., 2007).

After neutrophils are activated, they will synthesize a large number of immunomodulatory signals (Mariani et al., 2019): CXCL8 (the most significant chemokine, the main target of which is the neutrophil itself), CCL2 (C Chemokine ligands2), and CCL4. CCL2 and CCL4 are both effective chemotactic and activating factors for immune cells (Yamashiro et al., 2001). The gradual increase of these chemokines promotes the infiltration of monocytes and inhibits the infiltration of neutrophils. The lack of this signal cannot further activate neutrophils, enter the apoptotic pathway, and then be cleared from the site by phagocytes (Anderson et al., 2008). At the same time, circulating monocytes respond to chemotactic agents and bind to the fibrinogen in the temporary matrix of biological materials, thereby being activated (Shen et al., 2004) and differentiated into classical activation or “M1” macrophages (Mariani et al., 2019). The classification of these cells is based on the secretion of IL-1β, IL-6, TNF-α, chemokines (Jones et al., 2007; Mesure et al., 2010), and enzymes.

Macrophages induce invasion of inflammatory cells *via* CCL2, CCL4, and CXCL8 (Jones et al., 2007), and before experiencing the “frustrated” phagocytosis (because the biological material is too large), they try to decompose the biological materials by producing ROS and releasing degrading enzymes (Mariani et al., 2019). This pathway eventually leads to increased cytokine release (Underhill and Goodridge, 2012). Like the wound healing stage (Mariani et al., 2019), the adherent macrophages may eventually transfer to the “M2” phenotype (Mariani et al., 2019), which secrete IL-10 and so on (various anti-inflammatory cytokines). These cytokines can reduce their degradation ability, complete body activity reproduction, induce fibroblast movement and proliferation, and then achieve bone regeneration. Conversion between M1 and M2 and the mechanism of blocked phagocytosis leads to the fusion of macrophage membranes and the formation of foreign body giant cells (FBGCs), a sign of chronic inflammation on the coverage of biological materials. FBGCs formation is usually a landmark part of Foreign body reaction (FBR) induced by biological materials. It promotes the formation of FBR by activating mast cells, basophils, and Th cells. These cells can produce IL-4 and IL-13, enhancing the fusion of macrophages on biological materials (Brodbeck et al., 2005).

In the chronic inflammatory stage, some cytokines are secreted by Th cells. Their rich cytokines produce a wide range of regulation of pro-inflammatory or anti-inflammatory sequels (Mariani et al., 2019). The interactions between M1 and M2 macrophages and the changes of Th1 to Th2 cells express cell factors, indicating that T cells play a vital role in promoting the resolution and regeneration of inflammation.

The synergy of immune cells induces the production of pro-fibrotic factors, such as Platelet-derived growth factor (PDGF) (Shen et al., 2006), vascular endothelial growth factor (VEGF) (Chen et al., 2010), and TGF-β (Garg et al., 2009), which can recruit some fibroblasts. Experimental repair of damaged tissue, activated type I and type III collagen. Fibroblasts are responsible for accumulating new substrate, which is the process of fibrosis reaction.

Regarding the mechanism of promoting regeneration, M2 macrophages with anti-inflammatory/anti-fibrotic phenotypes promote regeneration through mutual interference with regulatory T cell subsets (Tregs) that play a vital part in the immune system. These cells regulate the tilt of the local immune response to inflammation, which further promotes the cascade of tissue regeneration and repair. Moreover, maintain anti-inflammatory and anti-fibrotic phenotypes by secreting IL-10. In addition, Tregs can enhance the ability to heal by inducing type 2 responses. After the reduction of T cells, the level of resident Tregs is still elevated, which may be because they lack epidermal growth factor receptor (EGFR) (Zaiss et al., 2013; Arpaia et al., 2015), whose expression can promote the cell factors secreting by mast cells to keep the Tregs in the damaged spot (Zaiss et al., 2013). Once Treg cells appear, they will proliferate and upregulate the secretion of bimodules, which is necessary for cell regeneration and may produce cell proliferation or induce cell differentiation. Treg cells may also enhance the regeneration ability of endogenous stem cells and progenitor cells through growth factors secret.

## 4 Immunomodulatory applications of biomaterials in skeletal disease

**FIGURE 5:**
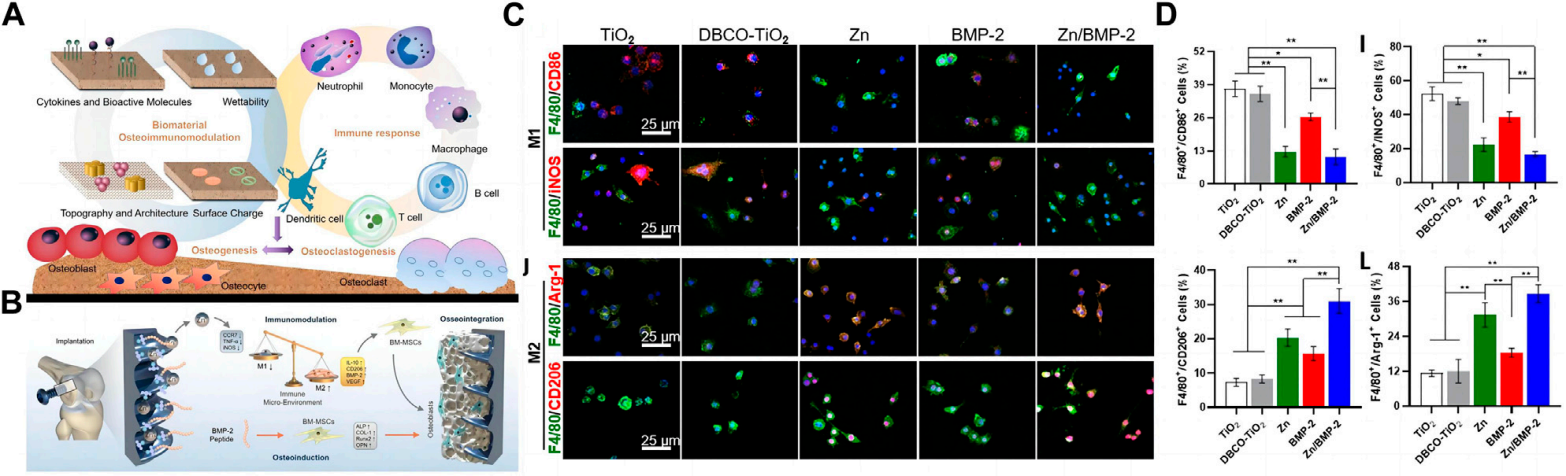
Immune cascade after bone implant material. **(A)** The interaction of biomaterials, skeletal cells, and immune cells, the graph was reprinted with permission from (Xie et al., 2020). **(B–D)** Zn^2+^ and BMP-2 peptide-modified titanium screws promote macrophage M2 polarization and synergize with BMP-2 to promote bone formation. The graph was reprinted with permission from (Wang et al., 2022).

The exploration of immunomodulation in biomaterials in regenerative medicine has attracted much attention. An in-depth understanding of materials’ immune cascade reaction principle (Xie et al., 2020) (Figure 5A) is critical in developing and innovating new materials.

### 4.1 The application of neutrophils in the design of biomaterials

Although neutrophils do not make up much in the bone marrow, they are the first cells to be mobilized in inflammatory and traumatic fractures. Neutrophils, pioneer cells of the inflammatory response, play a dual role in bone regeneration and repair. On the one hand, neutrophils eliminate cell debris, infectious substances, and phagocytic material scaffolds at the injury site by secreting proteases (e.g., matrix metalloproteinases, collagenases) and provide sites for subsequent bone regeneration. Cytokines such as IL-17 and TNF-α synthesized by neutrophils activate osteoclast through the RANKL-RANK signaling pathway (Chang et al., 2013). On the other hand, neutrophils stimulate angiogenesis (Christoffersson et al., 2012) and bone regeneration through direct or indirect action. Neutrophils in liver injury achieve angiogenesis and maturation by secreting MMP-9 to promote the expression of VEGF (Christoffersson et al., 2012). In the early stage of inflammation, neutrophils alleviate acute inflammation and reduce the secretion of inflammatory cytokines, thereby reducing the activation of osteoclast precursor cells and promoting bone repair (Bastian et al., 2018). The application of biologically active neutrophil membrane materials is enthusiastically sought after by scientific researchers. After implantation, the membrane material simulates the post-inflammatory process, and the apoptosis of neutrophils induces the entrapment of macrophages, which further promotes the polarization of macrophages to M2 to play an anti-inflammatory role and promotes bone regeneration. During this process, TGF-β and IL-10 secreted by macrophages promote anti-inflammatory and bone regeneration repair. IL-8-induced polarization of neutrophils contributes to endochondral ossification (Cai et al., 2021). These will provide strong support for designing materials to regulate neutrophil behavior.

### 4.2 The application of macrophages in the design of biomaterials

The application of macrophage polarization in the regeneration of bone defect sites is favored. The changes in macrophage behavior promote vascular and bone regeneration (Loi et al., 2016; ElHawary et al., 2021). Angiogenesis provides various nutrients and cells for bone repair. Therefore, coupling blood vessels and bone regeneration is another entry point for material design in fracture repair. The modified application of various bioactive molecules combined with biomaterials promotes vascular or bone regeneration by regulating macrophage polarization (Loi et al., 2016; Wang et al., 2022). Calcium silicate combined with β-tricalcium silicate stimulates the polarization of macrophages to M1/M2 by releasing IFN-γ and Si to secrete further vascular growth factors (VEGF, CXCL-12, PDGF-BB) to promote angiogenesis (Loi et al., 2016). Mesoporous silica (MNS)-loaded BMP bioactive molecules were incorporated into 3D printed scaffolds made of modified GelMA to release bioactive molecules and promote macrophage M2 polarization, BMP active molecules, and M2-secreted anti-inflammatory cells factors to promote the repair of diabetic bone defects. The release of BMP-2 and zinc particles regulates the increased proportion of M2 macrophages on the screw-coated surface to promote bone regeneration (Wang et al., 2022) (Figures 5B–D). Although macrophage polarization occurs in both angiogenesis and bone regeneration, there is a particular deviation in the macrophage’s temporal and spatial control time. It is urgent to develop new biomaterials to simultaneously realize the classification of the fate of macrophages in angiogenesis and bone regeneration.

### 4.3 The application of T cells in the design of biomaterials

**FIGURE 6:**
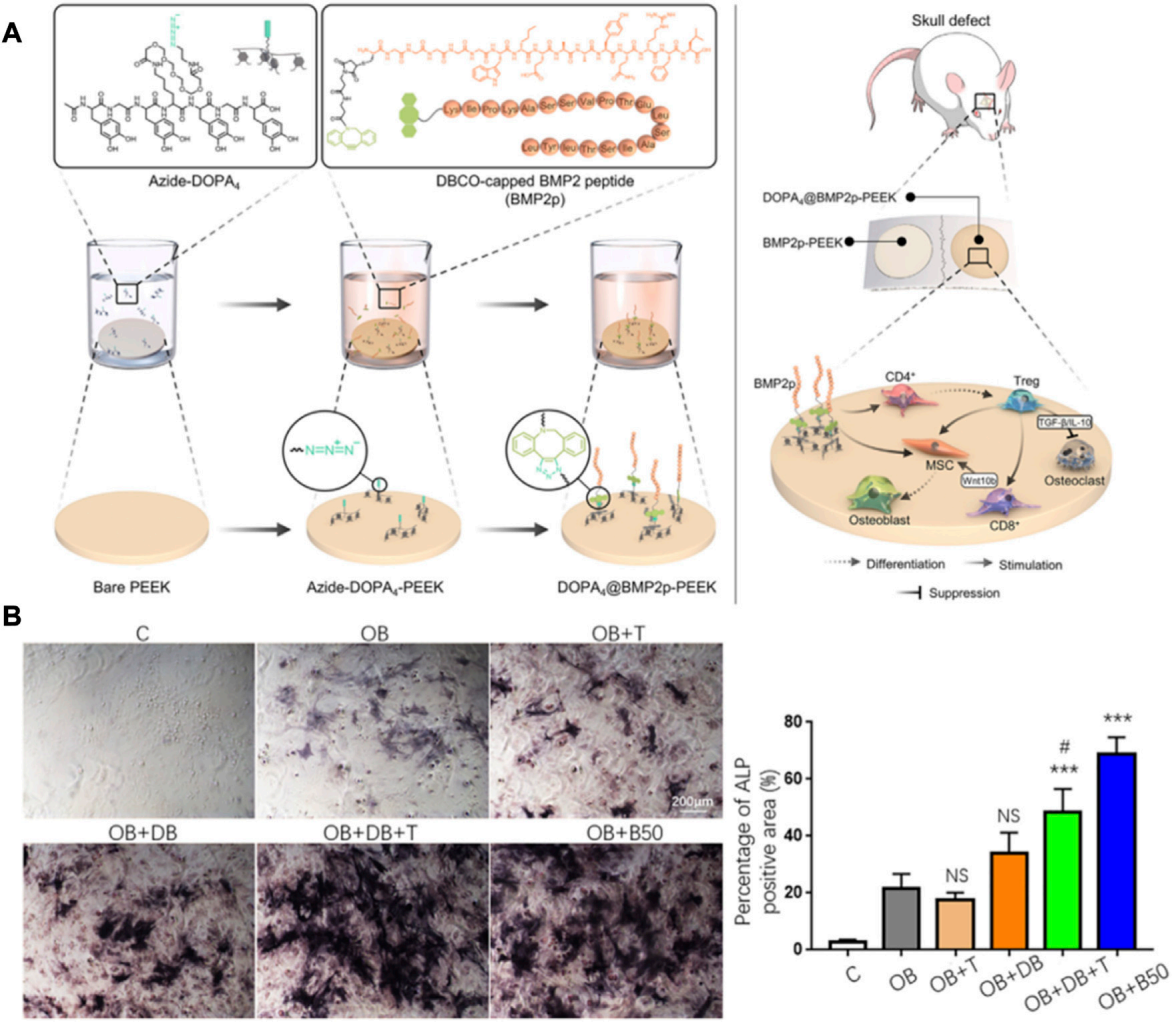
Immunomodulatory material for bone regeneration. **(A,B)** DOPA4@BMP2pPEEK material regulates the differentiation of CD4 T cells and synergizes with BMP-2 to promote bone regeneration. The graph was reprinted with permission from (Zhao et al., 2021c).

T cells play an indirect role in bone regeneration and repair, and factors secreted by various T cell subtypes indirectly regulate the process of bone regeneration. Like macrophages, T cells can be roughly divided into pro-inflammatory cell subtypes (Th1, Th17) and anti-inflammatory cell subtypes (Th2, Treg). Th1 cells can inhibit the expression of RUNX2 in BMSCs by secreting the IFN-γ factor to reduce bone repair ability (Liu et al., 2011). In addition, IFN-γ secreted by Th1 cells is associated with the polarization of M1 macrophages, and sustained M1 polarization leads to sustained inflammatory activation that prevents bone repair from occurring (Spiller et al., 2015). Th17 acts as the ability to secrete pro-inflammatory cytokine IL-17, but its role can promote bone regeneration and repair. IL-17A combined with BMP-2 can significantly enhance the osteogenic ability of BMSCs (Moon et al., 2016). Th2 and Treg cells promote bone homeostasis towards osteogenesis and bone regeneration by inducing the formation of M2 macrophages (Chen et al., 2012) and inhibiting osteoclast differentiation (Lu et al., 2017). And the surface of PEEK implants promotes bone regeneration by regulating T cell differentiation *via* lickable mussel-inspired azide-DOPA4 and BMP2p coupling (Zhao et al., 2021c) (Figure 6). Although there are few studies on T cells in bone regeneration biomaterials, the regulation of macrophages and osteoclasts by T cells will be a practical starting point for biomaterials to regulate the immune microenvironment to achieve bone regeneration and bone disease treatment. This will provide new therapeutic strategies for clinical bone defect regeneration and disease treatment.

## 5 Conclusion and future perspectives

The research of biomaterials is reviewed in bone regeneration and repair. We summarize some defects and improvement schemes of biomaterials and analyze the promising prospect of bone immune materials. It can be used for reference in the design of immune materials for bone regeneration.

1) Tricalcium phosphate ceramics (β-TCP) have been used as bone-filling materials in clinical practice due to their high biocompatibility and bone conductivity, but their non-degradability limits their application in bone regeneration. Then, biodegradable biomaterials with the same mechanical strength as β-TCP will be gradually explored. And the high biocompatibility and plasticity of the extracellular matrix (ECM) components will provide a broad application prospect for the research of bone regeneration materials.
2) Electrospun fibers’ tenacity, plasticity, and core-shell structure can carry hydrophilic and non-hydrophilic drugs, proteins, peptides, etc. They have been used as periosteal-like and filling delivery materials in bone defect models to promote bone regeneration. Still, the application of cytotoxic solvents in their synthesis process has not been able to promote their clinical application. The development of biological fibers will provide a new direction for the wide application of nanofibers.
3) 3D bio-printing materials have been widely used in the study of bone regeneration because of their ability to simulate organisms’ tissue structure accurately. Although it can print a material resembling bone, it can not respond to internal activity when implanted in organisms. The intelligent programming of 4D bio-printing materials and the function of responding to the activity of implants will provide broad application space for the research of bone regeneration.
4) M1 macrophages are important in clearing foreign bodies and dead cells at defect sites. They are indispensable in bone defect repair. Therefore, controlling the ratio of M1/M2 is the key to regeneration repair. In addition, how to transform macrophages from type M1 to type M2 after implantation is also the key to initiating the regenerative repair.
5) Numerous articles have focused on the effects of materials on macrophage behavior, with little focus on studies of other immune cells such as T cells, neutrophils, and DC cells. Different subtypes of T cells play multiple roles in the regeneration process, and neutrophils are also key cell populations that initiate the regenerative function of macrophages. The design of immunomodulatory materials targeting T cell subtypes and other immune cell behaviors may provide a new route for regenerative applications.

The success or failure of biomaterials in bone injury repair is determined by the interaction of the host immune system with biomaterials. Continued activation of the inflammatory response will lead to a delay in the repair process or tissue necrosis. Therefore, the rational regulation of the foreign body reaction effect is the key to bone injury regeneration and repair. From the selection of raw materials and the synthesis process, Biomaterials endowed with bioactive factors or their characteristics regulate immune cells, thus realizing the regulation of tissue regeneration. Competent biomaterials regulate the biological behavior of immune cells such as neutrophils, macrophages, or T cells. It promotes the differentiation of these immune cells to an anti-inflammatory phenotype, further promoting bone tissue regeneration. The research of new biomaterials to promote the real-time regulation of immune cell behavior to adapt to the high degree of compatibility between the immune system and bone regeneration, the tacit cooperation of these new biomaterials in innate and adaptive immunity needs to be further explored. Therefore, the design of biomaterials must consider the activation of immune cells and the mutual interference between different innate and adaptive cellular components to meet the research needs of clinical medicine.
